# Dorsal vein complex preserving technique for intrafascial nerve-sparing laparoscopic radical prostatectomy

**DOI:** 10.1111/j.1442-2042.2012.03181.x

**Published:** 2012-10-08

**Authors:** Akio Hoshi, Yukio Usui, Yuuki Shimizu, Tetsuro Tomonaga, Masayoshi Kawakami, Nobuyuki Nakajima, Kazuya Hanai, Takeshi Nomoto, Toshiro Terachi

**Affiliations:** Department of Urology, Tokai University School of MedicineIsehara, Kanagawa, Japan

**Keywords:** laparoscopic surgery, prostate cancer, quality of life, sexual function, urinary incontinence

## Abstract

**Objectives:**

To describe a novel dorsal vein complex preserving technique for intrafascial nerve-sparing laparoscopic radical prostatectomy and to evaluate its postoperative outcomes.

**Methods:**

A total of 109 patients who underwent laparoscopic radical prostatectomy by a single surgeon were evaluated, including 44 patients with dorsal vein complex preserving technique for intrafascial nerve-sparing laparoscopic radical prostatectomy, 20 patients with conventional intrafascial nerve-sparing laparoscopic radical prostatectomy and 45 patients with non-nerve-sparing laparoscopic radical prostatectomy. Functional outcomes were evaluated using a self-administered questionnaire (Expanded Prostate Cancer Index Composite). Continence was defined as zero to one security pad per day. Oncological outcomes were evaluated based on positive surgical margin.

**Results:**

In the dorsal vein complex preserving technique for intrafascial nerve-sparing laparoscopic radical prostatectomy group, the continence rate was 57%, 77% and 95% at 1, 3 and 12 months, respectively. The continence rate in the conventional intrafascial nerve-sparing laparoscopic radical prostatectomy group was 37%, 63% and 90%, and in the non-nerve-sparing laparoscopic radical prostatectomy group it was 23%, 57% and 82% at 1, 3, and 12 months, respectively. The dorsal vein complex preserving technique for intrafascial nerve-sparing laparoscopic radical prostatectomy group showed a significantly earlier recovery from incontinence compared with that in the conventional intrafascial nerve-sparing laparoscopic radical prostatectomy and non-nerve-sparing laparoscopic radical prostatectomy groups (log–rank test, *P* = 0.044 and *P* < 0.001). Similarly, the dorsal vein complex preserving technique for intrafascial nerve-sparing laparoscopic radical prostatectomy group tended to show a more early recovery in relation to urinary function of the Expanded Prostate Cancer Index Composite. Regarding sexual function, there were no significant differences between the dorsal vein complex preserving technique for intrafascial nerve-sparing laparoscopic radical prostatectomy and conventional intrafascial nerve-sparing laparoscopic radical prostatectomy groups. In pT2 patients, the positive surgical margin rate of the dorsal vein complex preserving technique for intrafascial nerve-sparing laparoscopic radical prostatectomy group (11%) was similar to that of the other two groups (conventional intrafascial nerve-sparing laparoscopic radical prostatectomy 7%; non-nerve-sparing laparoscopic radical prostatectomy 11%).

**Conclusions:**

The dorsal vein complex preserving technique for intrafascial nerve-sparing laparoscopic radical prostatectomy technique provides early recovery from incontinence without adversely affecting the oncological outcome.

## Introduction

The major postoperative complications after LRP include urinary incontinence and ED, especially in the early postoperative period. To improve these functional outcomes, the usefulness of LRP with the intrafascial nerve-sparing method has been recently reported.^1^–^5^ Because LRP provides a magnified field of view with less bleeding, there is a clear view of the microanatomy around the prostate. This makes it possible to carry out a delicate surgical procedure. In April 2008, we began using intrafascial nerve-sparing LRP to improve patients’ postoperative QOL. In addition, starting in January 2009, for earlier recovery from incontinence, we initiated a new “DVC preserving” technique. This DVC preserving technique involved preservation of the 12 o'clock position of the NVB. Therefore, this new technique involved wider preservation of the nerves and blood vessels distributed around the urethra than conventional intrafascial nerve-sparing techniques. In the current study, the perioperative functional and oncological outcomes of LRP using the DPLRP were evaluated.

## Methods

The present study included 109 patients who underwent LRP by a single surgeon (AH) at the Tokai University Hospital, Isehara, Kanagawa, Japan, between January 2009 and October 2011. Of the 109 patients, 44 had DPLRP carried out. Perioperative outcome, PSM rate, the postoperative continence rate and sexual function after DPLRP were retrospectively examined. Urinary and sexual outcomes were evaluated using a self-administrated QOL questionnaire; the EPIC and the IIEF-5. For continence rate, patients who answered “zero or one pad per day” on the EPIC (using question 5) were considered to be continent. As a control group, 20 patients who underwent CILRP and 45 patients who underwent NNLRP by the same surgeon during the same period were included.

### Indication for DPLRP

The basic indications for intrafascial nerve-sparing techniques (DPLRP and CILRP) were a PSA ≤10 ng/mL and stage ≤cT2a, without tumor foci in the nerve-sparing side, which was diagnosed by pathological results of biopsy and MRI findings. The technique of biopsy at our institution was 10 cores for the transrectal biopsy; eight cores collected from the peripheral-zone (four cores at the apex and four cores at the middle/base position) and two cores collected from the TZ. We carried out DPLRP when the patient had only one cancer focus. However, patients who had large TZ cancer (≥1 cm in diameter, using MRI) that was in contact with a DVC were excluded from the indication for DPLRP. Bilateral nerve-sparing DPLRP was carried out with selective cases, which included cT1 and only one cancer focus existed in the central part of the prostate.

### Statistical analysis

Continuous variables were compared with the Student's *t*-test or the two-tailed Fisher's exact test. Comparison of the interval to achieve continence was carried out with the log–rank test and Kaplan–Meier plots. Statistical significance was defined as *P* < 0.05.

### Surgical technique

**Fig. 1:**
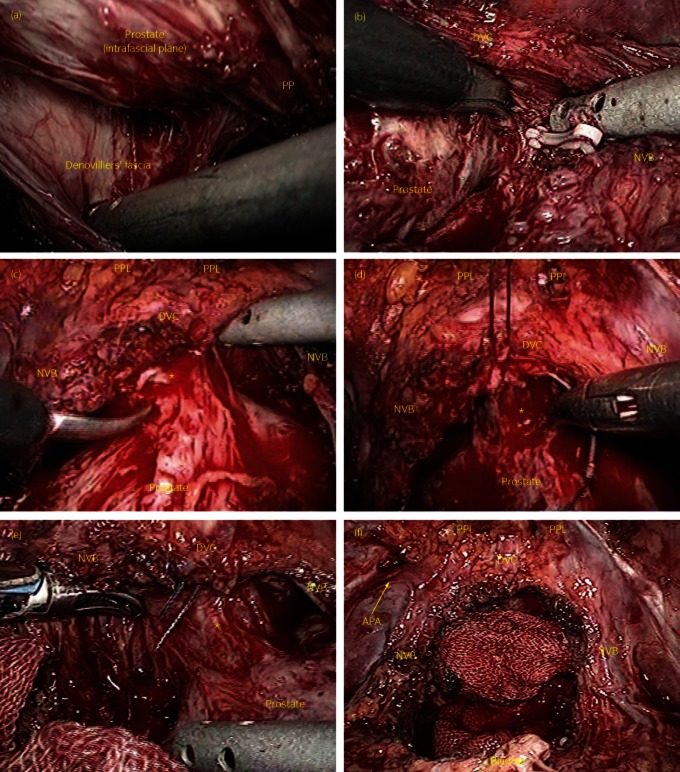
Intraoperative view of DPLRP (bilateral nerve-sparing). (a) Posterior prostate dissection in the intrafascial plane and prostatic pedicle dissection. (b) Lateral to anterior prostate dissection in the intrafascial plane and wide sparing of the neurovascular bundle. On the nerve-sparing side, Hem-O-lock clips were used to achieve hemostasis in the prostatic pedicles. An assistant lifted the widely-preserved NVB and DVC, and provided appropriate counter traction to ensure the field of view. (c) Dissection of the apex and detachment of the dorsal vein complex from the prostate by cold cutting with scissors. (d,e) A stay suture was placed at the 12 o'clock position of the DVC for hemostasis from the DVC. (f) Wide preservation of the dorsal vein complex, neurovascular bundle, puboprostatic ligaments and apical accessory pudendal artery.

First, bladder neck dissection was carried out where a 1.5-cm transverse incision was made in the visceral endopelvic fascia above the bladder neck. From this window of fascia, the bladder neck was bluntly dissected to expose the urethra using the bladder neck preservation technique. After the urethra was cut, the seminal vesicles and the ampulla of vas deferens were identified and dissected. On the nerve-sparing side, Denonvilliers’ fascia was preserved and stripped down from the prostatic capsule at the posterior surface of the prostate. From this posterior surface, the appropriate layer of the intrafascial plane was easily peeled off (Fig. 1a). In this intrafascial plane, we carried out careful sharp and blunt dissection using cold scissors at the lateral aspect of the prostate, the prostatic pedicles were controlled, and the dissection proceeded from the posterior to the apex and anterior surface of the prostate. In this area, Hem-O-lock clips were used to achieve hemostasis in the prostatic pedicles, and thermocoagulation was not used as much as possible to prevent thermal damage of the pelvic plexus and NVB (Fig. 1b).^2010^

At the apex, we carried out DVC non-ligating and puboprostatic ligament preservation methods. Even on the non-sparing side, the puboprostatic ligament including the APA was also preserved. On the anterior surface of the apex, the anterior dissection was carried out with scissors to separate DVC from anterior fibromuscular stroma of the prostate (Fig. 1c).^2010^ Additionally, a stay suture for hemostasis was placed at the 12 o'clock position of the DVC using a 3-0 braided-lactomer suture on a CV-25 needle (Fig. 1d,e). With the aforementioned technique, the pelvic floor structures including the nerves and blood vessels (e.g. NVB, APA and DVC) were widely preserved (Fig. 1f). After bladder neck formation with everting-sutures, the posterior wall (posterior prostatic fascia) was reconstructed using one 20-cm 3-0 braided-lactomer suture on a CV-25 needle. Urethrovesical anastomosis was carried out using the running-suture method with two 20-cm 3-0 braided-lactomers on a GU-46 needle. In addition, the anterior wall (anterior detrusor apron) was reconstructed after anastomosis. In the present study, the posterior and anterior wall reconstruction technique was carried out in all patients.

## Results

There was no significant difference in each group with respect to age, clinical stage and biopsy Gleason score (Table 1). PSA level was significantly different between the DPLRP and NNLRP group. Seven patients (15.9%) in the DPLRP group and three patients (15%) in the CILRP group underwent bilateral nerve-sparing LRP. There was no statistically significant difference between the groups with respect to the proportion of nerve-sparing procedures (Table 1). There was also no significant difference with respect to operative time, postoperative complication and postoperation hospital stay (Table 1). The median estimated blood loss including urine in the DPLRP group was greater than that in the NNLRP group (*P* = 0.009). In all three groups, there were no intraoperative complications and no patients required allogeneic blood transfusion during and after surgery. Postoperative complications requiring surgical treatment occurred in only one patient with lymphorrhea who underwent DPLRP (Table 1).

**Table 1:** Patient characteristics of the DPLRP, CILRP and NNLRP groups

	DPLRP *n* = 44	CILRP *n* = 20	NNLRP *n* = 45	*P*-value (*vs* CILRP)	*P*-value (*vs* NNLRP)
Mean age (years)	65.4 ± 6.8	66.5 ± 5.4	65.7 ± 5.3	0.556	0.818
PSA, mean ± SD (ng/dL)	7.15 ± 2.6	6.23 ± 2.0	10.2 ± 6.3	0.176	0.004
Clinical T stage				0.985	0.412
cT1	28 (63.6%)	13 (65.0%)	23 (51.1%)		
cT2	16 (36.4%)	7 (35.0%)	19 (42.2%)		
cT3	0	0	3 (6.7%)		
Biopsy Gleason score				0.556	0.993
6	18 (40.9%)	12 (60%)	18 (40%)		
7	20 (45.5%)	6 (30%)	21 (46.7%)		
≥8	6 (13.6%)	2 (10%)	6 (13.3%)		
Bilateral nerve sparing	7 (15.9%)	3 (15%)	–	0.781	–
Median operative time, min (range)	268.5 (171–364)	289.5 (178–387)	250 (138–345)	0.634	0.185
Median estimated blood loss including urine, mL (range)	407 (55–1438)	378.5 (69–731)	206 (75–996)	0.568	0.009
Postoperative complications	1 (2.3%)	0	0	0.683	0.991
Median post operation hospital stay, days (range)	7 (5–17)	7 (5–13)	7 (5–14)	0.354	0.325

### Postoperative pathological results

All three groups had no significant differences in their pathological stage, in the pathological Gleason score, in the frequency of the PSM and in the location of PSM (Table 2). The PSM in the DPLRP group was located in the ventral TZ in 37.5%, the apex in 37.5% and was posterolateral in 25% of patients. Three patients (37.5% of PSM patients underwent DPLRP) had PSM on the nerve-sparing side. In the DPLRP group, the PSM was more common on the ventral TZ of the nerve-sparing side than in the other regions. There tended to be a higher proportion of the PSM on the ventral TZ in the DPLRP (37.5%) group than in the CILRP (0%) and NNLRP groups (0%); however, this was not statistically significant (Table 2).

**Table 2:** Postoperative pathological results

	DPLRP *n* = 44	CILRP *n* = 20	NNLRP *n* = 45	*P*-value (*vs* CILRP)	*P*-value (*vs* NNLRP)
Pathological T stage				0.769	0.959
pT2	36 (81.8%)	15 (75%)	36 (80%)		
pT3	8 (18.2%)	5 (25%)	9 (20%)		
Pathological Gleason score				0.879	0.961
6	16 (36.4%)	9 (45%)	18 (40%)		
7	22 (50%)	8 (40%)	21 (46.7%)		
≥8	6 (13.6%)	3 (15%)	6 (13.3%)		
Positive surgical margin rate					
pT2	11.1% (4/36)	6.7% (1/15)	11.1% (4/36)	0.975	0.709
pT3	50% (4/8)	100% (5/5)	33.3% (3/9)	0.12	0.839
Overall	18.2% (8/44)	30% (6/20)	15.6% (7/45)	0.463	0.796
Location of positive surgical margins				0.7845	0.528
Apex	37.5% (3/8)	33.3% (2/6)	85.7% (6/7)		
Posterolateral	25% (2/8)	50% (3/6)	0		
Ventral (transition zone)	37.5% (3/8)	0	0		
Bladder side	0	16.7% (1/6)	14.3% (1/7)		
Overall	8	6	7		

### Functional outcomes

**Fig. 2:**
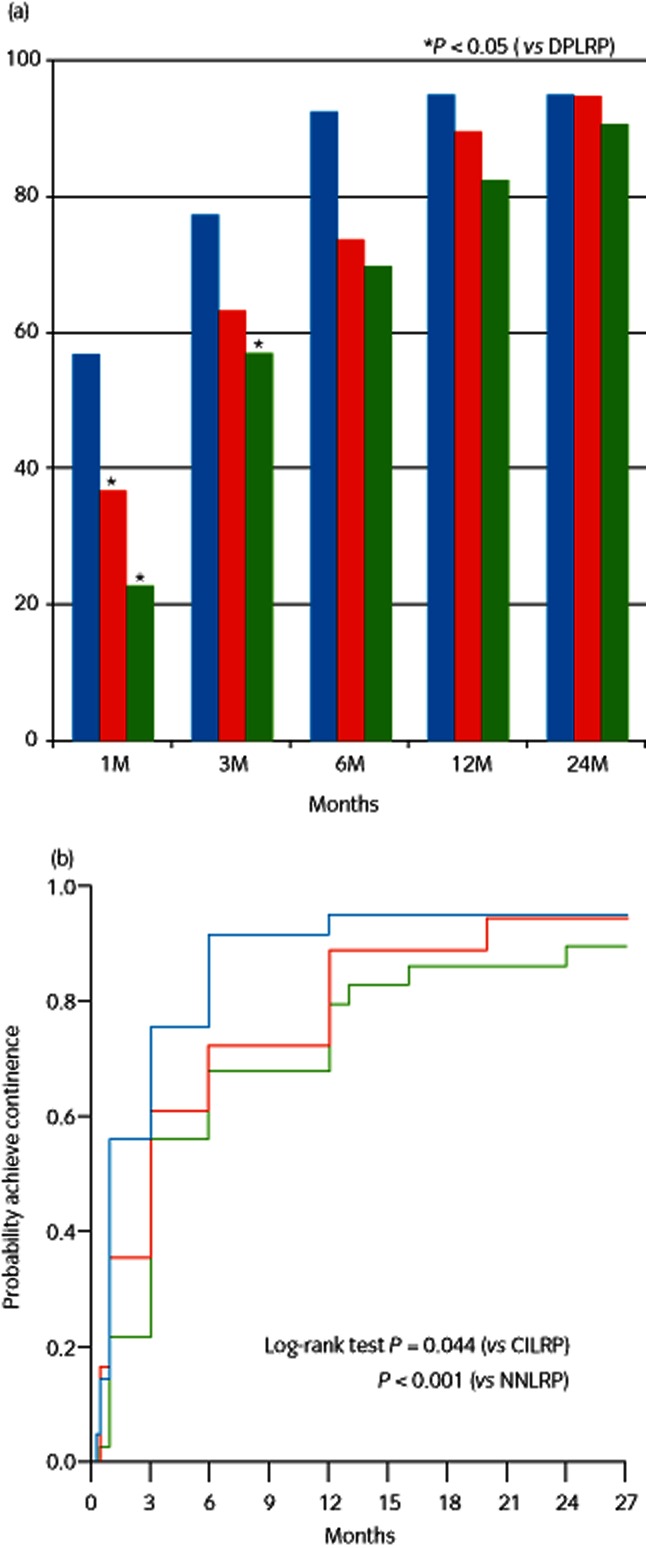
Urinary outcomes after DPLRP, CILRP and NNLRP. (a) Continence rate after LRP by months after surgery for the DPLRP, CILRP and NNLRP groups. (b) Kaplan–Meier curves show the probability of achieving continence after LRP for the DPLRP, CILRP and NNLRP groups. Asterisks indicate *P* < 0.05 (*vs* DPLRP). 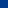, DPLRP; 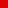, CILRP; 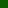, NNLRP; 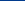, DPLRP; 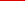, CILRP; 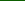, NNLRP.

With regard to the continence rate, in the DPLRP group, the postoperative continence rate was 56.8%, 77.3%, 92.4%, 94.9% and 95% at 1, 3, 6, 12 and 24 months, respectively. In the CILRP group, the postoperative continence rate was 36.8%, 63.2%, 73.7%, 89.5% and 94.7%, and in the NNLRP group it was 22.7%, 56.8%, 69.5%, 82.2% and 90.5% at 1, 3, 6, 12 and 24 months, respectively. The continence rate for the DPLRP group was significantly higher than that for the CILRP and NNLRP groups at 1 month (*P* = 0.029 and *P* = 0.001), and it was also higher than that for the NNLRP group at 3 months (*P* = 0.041) (Fig. 2a). In addition, we investigated the probability of achieving continence using Kaplan–Meier plots (Fig. 2b). The median interval to achieve continence was 1 month in the DPLRP group, and 3 months in the CILRP and NNLRP groups. The mean interval to achieve continence was 3 months in the DPLRP group, 5.8 months in the CILRP group and 7.8 months in the NNLRP group. The probability of achieving continence was significantly higher in the DPLRP group compared with the CILRP and NNLRP groups (log–rank test, *P* = 0.044 and *P* < 0.001) (Fig. 2b).

**Fig. 3:**
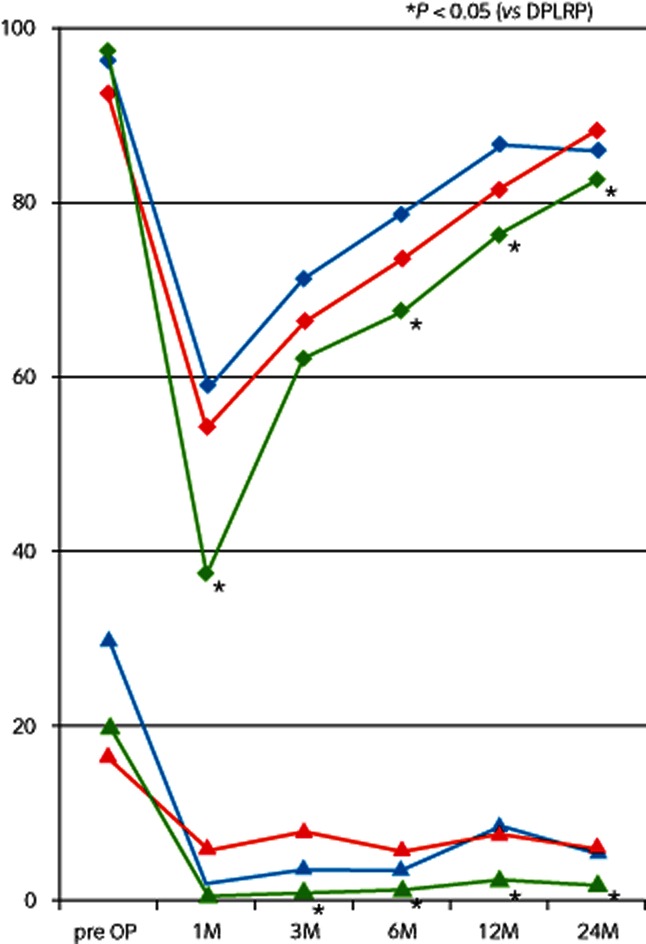
Urinary and sexual function scores of the EPIC. Asterisks indicate *P* < 0.05 (*vs* DPLRP). UF = mean urinary function score of the EPIC, SF = mean sexual function score of the EPIC. 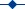, UF of DPLRP; 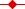, UF of CILRP; 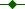, UF of NNLRP; 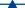, SF of DPLRP; 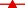, SF of CILRP; 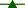, SF of NNLRP.

For urinary function of the EPIC score, the DPLRP group showed a significantly earlier recovery compared with urinary function in the NNLRP group at 1, 6, 12 and 24 months (*P* < 0.05, Fig. 3); however, there was no significant difference compared with the CILRP group (Fig. 3).

Regarding sexual function, there were no significant differences among all groups in preoperative sexual function score of the EPIC. Comparison of postoperative recovery between the DPLRP and NNLRP groups showed that sexual function was significantly higher for the DPLRP group at 3, 6, 12 and 24 months (*P* < 0.05, Fig. 3). However, there was no significant difference among the nerve-sparing groups in sexual function after LRP (Fig. 3). With regard to the results of the IIEF-5 score, the same tendency for sexual function was observed.

## Discussion

Early recovery from incontinence and ED after radical prostatectomy is important for postoperative QOL, and this is equally as important as cancer control. The knowledge of microanatomy is defined as “fascia” including nerves and blood vessels distributed around the prostate and urethra.^7^–^11^ This knowledge has resulted in the new concept of “intrafascial dissection”, which was not previously considered in radical prostatectomy.^1^,^12^,^13^ In addition, the usefulness of LRP with the modification of apical dissection and control DVC methods has been recently reported.^14^,^15^ Based on these concepts, new techniques have improved for cancer control and functional outcomes.^1^,^5^,^15^,^16^ The aim of DPLRP is to improve functional outcome by preserving the dominant nerves and blood vessels distributed around the urethra and by preserving and maintaining pelvic floor structures.

Our DPLRP technique was modified based on the “Veil technique” during RALP, as reported by Menon *et al*.^2007^ using a widely-spared NVB except for the DVC. In the DPLRP technique, dissection into the intrafascial plane is initiated on the posterior surface of the prostate using the antegrade approach, as reported by Menon *et al*.^2007^ Similar methods based on the same nerve-sparing concept have been reported as “intrafascial nerve-sparing” by Stolzenburg *et al*.^2008^ and “curtain dissection” by Lunacek *et al*.^2005^ These other methods are different compared with DPLRP. Stolzenburg *et al*.^2008^ and Lunacek *et al*.^2005^ carried out the retrograde approach, where dissection into the appropriate intrafascial plane was initiated on the anterior surface of the prostate at the 10 or 2 o'clock position. In addition, wide periprostatic tissue preservation including the DVC during RALP was recently reported by Asimakopoulos *et al*. as “complete periprostatic anatomy preservation”.^2011^ Although this preservation technique is the same concept as our DPLRP technique, the major difference in surgical technique is the dissection method into the intrafascial plane. Asimakopoulos *et al*. reported that the surgeon enters the intrafascial plane at the 2 o'clock position and rolls off the lateral surface of the mid prostate using the lateral approach. Anterior dissection of the pubovesical complex (i.e. the detrusor apron and DVC) is then carried out to spare both structures. There might be some difficulty when this preservation technique is carried out during LRP; for example, technical difficulty in carrying out vesicourethral anastomosis is expected. In addition, the seminal vesicle-sparing technique is included in complete periprostatic anatomy preservation.^2010^ In contrast, in our LRP technique, seminal vesicles are resected (not spared) with the prostate.

With regard to urinary outcomes after DPLRP, we found that the early continence rate for the DPLRP group was significantly higher than that for the CILRP and NNLRP groups (Fig. 2a,b). The median and mean intervals of continence were significantly shorter in the DPLRP group compared with those in the CILRP and NNLRP groups (Fig. 2b). Therefore, DPLRP was useful for early recovery from postoperative incontinence compared with the CILRP and NNLRP groups. In addition, the present results of the EPIC showed the efficacy of DPLRP in postoperative urinary function compared with NNLRP. A similar tendency was observed when DPLRP was compared with CILRP, although there was no significant difference between the groups (Fig. 3). Therefore, our data show that wider preservation of nerves and blood vessels achieves a much earlier recovery from postoperative incontinence and urinary dysfunction.

In our current study of unilateral nerve-sparing, which accounted for most cases (84%), continence rates of 56.8% at 1 month after DPLRP were comparable with those of 43–64% at 1 month after bilateral nerve-sparing LRP^5^,^18^–^20^ and 36–56% at 1 month after RALP with a variety nerve-sparing conditions.^3^,^21^,^22^ For early recovery from postoperative incontinence, we believe that preservation of nerves and blood vessels, such as the NVB, APA and DVC, is important. Furthermore, the present study included unilateral and bilateral nerve-sparing cases. The number of patients who underwent bilateral nerve-sparing was small, and the proportions of bilateral nerve-sparing cases were similar between the DPLRP and CILRP groups (Table 1). Therefore, we considered the bias in the status of nerve sparing to be limited.

In contrast, with regard to recovery of postoperative sexual function, there was no significant difference between the DPLRP and CILRP groups. It appears that a low preoperative sexual function was a major reason why recovery of sexual function after nerve-sparing LRP was not adequate. In the present study, just 9% of patients had normal preoperative sexual function (IIEF-5 score ≥22); 13.5% for DPLRP, 18.2% for CILRP and 0% for NNLRP. Notably, percentages of moderate to severe preoperative ED (IIEF-5 score ≤11) were 62.2% for DPLRP, 45.5% for CILRP and 43.3% for NNLRP. Consequently, approximately half of the patients in the present study had preoperative ED. The effect of DPLRP on sexual function should be further examined in a larger study of patients with normal to high preoperative sexual function.

For evaluating oncological outcome of LRP, we used the PSM rate, because the postoperative observation period was short (22.3 months) in the present study. In DPLRP, because periprostatic tissue (nerves and blood vessels) is widely preserved, an increased PSM rate is concerning, especially in pT2 cases. However, in the present study, the PSM rate in the DPLRP group was similar to that in the other groups in both pT2 and pT3 cases. Although the number of patients was small, our data suggest that the DPLRP technique does not increase the PSM rate, especially in pT2 cases. When we compared DPLRP with other techniques at our institution, the PSM rate for pT2 in 329 patients who underwent LRP by multiple surgeons was 18.3%. A PSM rate of 4.5–21.8% in pT2 cases has been reported by other authors,^1^,^20^,^23^–^26^ which is similar to our PSM rate for DPLRP in pT2 cases.

With regard to the location of PSM, the PSM in the DPLRP group was most often observed in the ventral TZ. In the other two groups, however, the PSM was most frequently located in the apex. Among the aforementioned 329 LRP cases at our institution, in 78 cases with PSM, the location of the PSM was the apex in 67% and the ventral TZ in just 4% of patients. The PSM rate of the DPLRP group in the ventral TZ was also higher than that in the 78 cases. Therefore, in our opinion, the PSM of the DPLRP group tended to be in high proportion if cancer foci were present in the ventral TZ. Although all patients who underwent DPLRP had preoperative MRI, there were no cancer foci detected in the TZ. It might be difficult to preoperatively diagnose cancer foci using preoperative imaging, especially in the ventral TZ. To improve PSM rates, adequate selection with a more accurate preoperative diagnosis are necessary. Alternatively, in cases where cancer foci have contact with the DVC, non-DVC preserving methods, such as CILRP, should be selected rather than DPLRP.

DPLRP provides earlier recovery of functional outcomes, especially postoperative incontinence without exacerbation of oncological outcome. However, there are some problems in surgical techniques with the DVC preservation technique; for example, widely-preserved NVB are prone to bleeding, which results in a narrow view, and these make urethrovesical anastomosis difficult. Therefore, DPLRP tends to have a greater estimated blood loss than conventional LRP. These technical problems can gradually be resolved with further case experience. The development of new equipment (e.g. 3-D imaging and energy devices) and further introduction of RALP should resolve these technical problems, and intrafascial nerve-sparing LRP, including DPLRP, will become more widespread.

The DPLRP technique provides significantly earlier recovery from postoperative incontinence compared with the CILRP and NNLRP techniques. However, postoperative sexual function after DPLRP is equivalent compared with that with CILRP. With regard to oncological outcome, the DPLRP technique is not associated with exacerbation of the PSM rate. Therefore, we conclude that the DPLRP technique is one useful method in nerve-sparing LRP.

## Abbreviations

APA: accessory pudendal artery
CILRP: conventional intrafascial nerve-sparing laparoscopic radical prostatectomy
DPLRP: dorsal vein complex preserving technique for intrafascial nerve-sparing laparoscopic radical prostatectomy
DVC: dorsal vein complex
ED: erectile dysfunction
EPIC: Expanded Prostate Cancer Index Composite
IIEF-5: International Index of Erectile Function-5
LRP: laparoscopic radical prostatectomy
MRI: magnetic resonance imaging
NNLRP: non-nerve-sparing laparoscopic radical prostatectomy
NVB: neurovascular bundle
PP: prostatic pedicle
PPL: puboprostatic ligament
PSA: prostate-specific antigen
PSM: positive surgical margin
QOL: quality of life
RALP: robot-assisted radical prostatectomy
SF: mean sexual function score of the Expanded Prostate Cancer Index Composite
TZ: transition zone
UF: mean urinary function score of the Expanded Prostate Cancer Index Composite
